# Hemophagocytic lymphohistiocytosis—how common and how severe is it as a complication of malaria? Retrospective case series and review of the literature

**DOI:** 10.1007/s15010-023-02104-w

**Published:** 2023-10-24

**Authors:** Hans Martin Orth, Dorothea Wiemer, Sophie Schneitler, Andreas Schönfeld, Martha Charlotte Holtfreter, Smaranda Gliga, Andre Fuchs, Frieder Pfäfflin, Claudia Maria Denkinger, Sven Kalbitz, Carlos Fritzsche, Marc P. Hübner, Janina Trauth, Björn-Erik Ole Jensen, Tom Luedde, Torsten Feldt

**Affiliations:** 1 Department of Gastroenterology, Hepatology and Infectious Diseases, Medical Faculty and University Hospital Düsseldorf, Heinrich Heine University, Düsseldorf, Germany; 2 Department of Tropical Medicine and Infectious Diseases at the Bernhard Nocht Institute, Bundeswehr Hospital Hamburg, Hamburg, Germany; 3 Institute of Medical Microbiology and Hygiene, Saarland University, Homburg/Saar, Germany; 4 Department of Infectious Diseases, West German Centre of Infectious Diseases, University Hospital Centre Essen, University of Duisburg-Essen DE, Essen, Germany; 5 Internal Medicine III-Gastroenterology and Infectious Diseases, Augsburg University Hospital, Augsburg, Germany; 6 Department for Infectious Diseases and Respiratory Medicine, Charité-Universitätsmedizin Berlin, Corporate Member of Freie Universität Berlin, Humboldt-Universität zu Berlin, and Berlin Institute of Health, Augustenburger Platz 1, 13353 Berlin, Germany; 7 Division of Infectious Disease and Tropical Medicine, Heidelberg University Hospital, Heidelberg, Germany; 8 German Center for Infection Research (DZIF), partner site Heidelberg University Hospital, Heidelberg, Germany; 9 Department of Infectious Diseases/Tropical Medicine, Nephrology and Rheumatology, Hospital St. Georg, Leipzig, Germany; 10 Department of Tropical Medicine and Infectious Diseases, Center for Internal Medicine, University of Rostock, Rostock, Germany; 11 Institute for Medical Microbiology, Immunology and Parasitology, University Hospital Bonn, Bonn, Germany; 12 German Center for Infection Research (DZIF), Partner Site Bonn-Cologne, Bonn, Germany; 13 Division of Infectious Diseases, Department of Internal Medicine II, University Hospital Giessen and Marburg, Justus Liebig University Giessen, Giessen, Germany

**Keywords:** Malaria, HScore, HLH, *Plasmodium*

## Abstract

**Background:**

Infection-associated secondary hemophagocytic lymphohistiocytosis (sHLH) is a potentially life-threatening hyperinflammatory condition caused by various infectious diseases. Malaria has rarely been described as trigger. The aim of this study is to collect data on frequency, clinical spectrum, and outcome of sHLH induced by malaria.

**Methods:**

We collected case numbers on malaria and malaria-associated sHLH from specialized centers in Germany from 2015 to 2022. In addition, we conducted a literature search on published cases of malaria-associated sHLH and systematically analyzed the literature regarding clinical and diagnostic criteria.

**Results:**

We obtained data from 13 centers treating 1461 malaria cases with different *Plasmodium* species, of which 5 patients (0.34%) also were diagnosed with sHLH. The literature search revealed detailed case reports from further 51 patients and case series comprising the description of further 24 patients with malaria-associated sHLH. Most cases (48/80; 60%) were reported from Asia. The median time interval between onset of malaria symptoms and hospital admission was 7 days. Severe complications of sHLH were documented in 36% (20/56) of patients, including two patients with multiple organ failure in our case series. Only 41% (23/56) of patients received specific treatment for sHLH, nevertheless the mortality rate (CFR) of 5% is lower compared to the CFR reported for sHLH triggered by other infectious diseases (e.g., 25% in sHLH due to EBV infection).

**Conclusion:**

Malaria-associated sHLH appears to have a comparatively good prognosis but may still represent an underdiagnosed and potentially fatal complication of malaria, especially in resource-poor settings.

**Supplementary Information:**

The online version contains supplementary material available at 10.1007/s15010-023-02104-w.

## Introduction

**Fig. 1:**
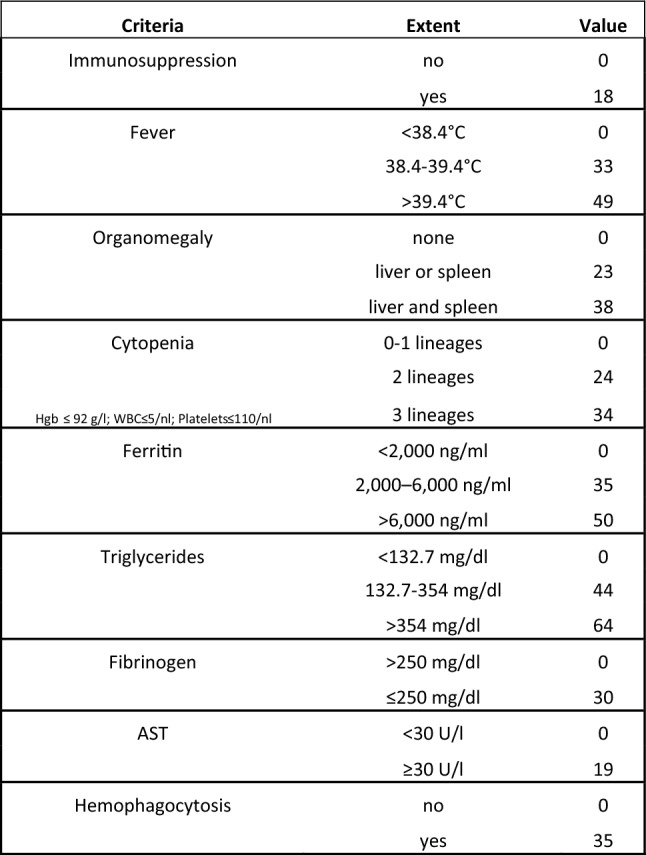
Calculation of HScore for the diagnosis of reactive hemophagocytic lymphohistiocytosis. The HScore is formed from the sum of the values of the individual criteria. *Hgb* hemoglobin, *WBC* white blood count, *AST* aspartate aminotransferase

**Fig. 2:**
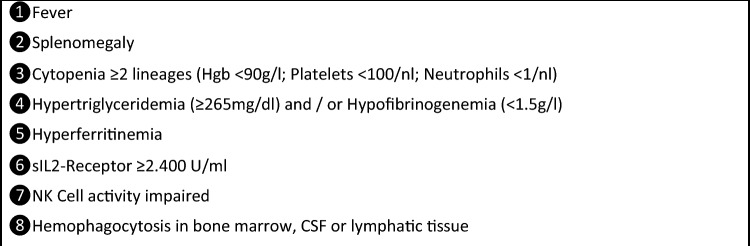
HLH-2004 criteria for the diagnosis of hemophagocytic lymphohistiocytosis. The diagnosis is confirmed if five or more criteria are fulfilled. *Hgb* hemoglobin, *sIL2* soluble interleukin-2, *CSF* cerebrospinal fluid

Hemophagocytic lymphohistiocytosis (HLH, formerly also named hemophagocytic syndrome) has first been described in 1939 by Scott and Robb-Smith as histiocytic medullary reticulosis [1]. Since then, the diagnosis has been subdivided into several groups, namely primary (genetic) HLH, and various secondary forms of HLH including infection-associated, malignancy-associated and autoimmune-associated HLH. When associated with rheumatic disease, HLH is often referred to as “macrophage activation syndrome” [2]. All entities share a hyperinflammatory condition with often vastly elevated ferritin levels. While genetic causes underlying primary HLH have been identified, no genetic risk factors for development of secondary HLH (sHLH) have ever been conclusively described. The eponymous hemophagocytosis is often visible in bone marrow aspirates, splenic and lymphatic tissue, but may also be absent. Diagnostic criteria (HLH-2004) have been established for a hereditary form of HLH and last updated in 2007 (see Fig. 1) [3]. These diagnostic criteria are commonly used for sHLH as well, but in 2014, the HScore was established as specific diagnostic algorithm for sHLH. The HScore is a sum of nine numerical values, each corresponding to individual diagnostic criteria (see Fig. 2). A cutoff of 169 has been shown to correctly classify 90% of all cases. Figures 1 and 2 list the diagnostic criteria of HScore in comparison to HLH-2004. As an advantage over the HLH-2004 criteria, the HScore does not require results for soluble interleukin-2 (sIL2) receptor and NK cell activity, which are not widely available even in high income countries. The disadvantage, however, is that all nine variables are required for a reliable statement: missing data cause falsely low results [4].

Infection-associated sHLH has first been described in 1979 [5]. Although commonly associated with viral infections (primarily Epstein–Barr virus, EBV), sHLH can also be triggered by other infectious agents, including bacteria, fungi, protozoa, and helminths, but mostly by agents that are capable of causing chronic or long-lasting infections. The causative pathogen also seems to determine the prognosis, with the worst prognosis documented in sHLH due to EBV infection, which has only a 75% survival rate even with optimal treatment [6]. Only few case reports mention malaria as an infectious trigger for HLH. We present five cases (four previously unpublished) of HLH in patients with malaria who were treated in infectious diseases and tropical medicine institutions in Germany. We also review clinical characteristics of 52 previously published cases of HLH secondary to malaria and attempt to estimate the risk of HLH following malaria using statistical data.

## Methods

This case series was compiled retrospectively from all patients reported by participating centers in Germany with confirmed sHLH after infection with *Plasmodium* species. All examinations and treatments were performed as part of the routine care. Data processing was conducted in compliance with good clinical practice and applicable regulations. Ethical approval was obtained from the respective institutional review boards or ethical committees.

### Patient data collection

We asked 27 specialized departments of tropical medicine and infectious diseases listed by the German Society for Tropical Medicine, Travel Medicine and Global Health (DTG) and the German Society for Infectious Diseases (DGI) to participate in a survey of patients treated with malaria who developed sHLH. Departments willing to participate were sent standardized questionnaires to collect the number of patients treated with malaria and data on each patient treated with sHLH due to malaria between 2015 and 2022.

### Literature research

On 2023/02/28, we conducted a PubMed search using the search string “(Malaria OR *Plasmodium*) AND (hemophagocytic syndrome OR haemophagocytic syndrome OR hemophagocytic lymphohistiocytosis OR haemophagocytic lymphohistiocytosis OR hemophagocytosis OR haemophagocytosis OR macrophage activation syndrome)”. Included were publications that contained case descriptions of HLH associated with malaria infection. Cases without access to full text were considered if a summary with relevant data was available. No restriction was applied regarding publication year or language. Articles that were not identified via the PubMed search but were found in the references of the returned articles were also considered if they met the above criteria.

All case reports were reviewed for fulfillment of diagnostic criteria and statistically evaluated for etiology, therapy, and outcome. To investigate the applicability of the newer HScore, we conducted calculations based on available data. In case of incomplete data, we assumed no presence of immunosuppression if not explicitly mentioned. According to common definitions, we assumed a temperature > 39.4 °C when the term “high-grade fever” was mentioned, and a temperature between 38.4 °C and 39.4 °C if fever was mentioned but not further specified [7].

### Statistical analysis

Descriptive analysis was performed using IBM SPSS Statistics for Windows, Version 21 (IBM Corp., Armonk, NY, USA). In cases where data for calculation of the HScore are incomplete, we decided against imputation and instead used the minimum and maximum possible value (assuming that either none or all the unnamed criteria were met). After consulting a statistician, we refrained from a multivariate analysis of risks for severe or fatal courses in view of the heterogeneous data sets of the individual case reports.

For risk assessment, we obtained statistical data on the reported number of malaria cases imported to Germany. To screen for additional sHLH cases triggered by malaria, we conducted a data bank survey at the Federal Statistical Office of Germany (Destatis), a governmental authority for statistical analysis. The data bank request was conducted for inpatients treated with any combination of the ICD-10 codes B50 to B54 (malaria) and D76.1/D76.2 (HLH) during a single hospital stay within the 6-year period from 2015 to 2020.

## Results

### Patients

Thirteen of the twenty-seven contacted institutions provided the requested data. During the defined 8-year period, these institutions had treated 1461 cases of malaria, and among those, 5 patients additionally met diagnostic criteria for HLH. One of these cases has been published previously [8].

In Germany, reporting malaria cases to the Robert Koch Institute (RKI), the German public health institute, is mandatory, and data are regularly published online [9]. According to RKI, a total of 6682 malaria cases had been reported in Germany during the selected period (2015: 1068; 2016: 970; 2017: 957; 2018: 896; 2019: 999; 2020: 366; 2021: 613; 2022: 813). We can, therefore, provide data for 21.9% (1461/6,682) of the reported malaria cases.

### Literature review

Our PubMed search yielded a total of 79 results with 45 case reports on 47 patients, including the above-mentioned patient whose case report we had already published [8, 10–53]. References of these articles comprised reports on five more cases which were included in the analysis [54–58].

Seven references were found that mentioned further twenty-four malaria-associated sHLH cases in a different context, mostly larger case series on infection-associated sHLH regardless of the causative agent. These publications did not contain specific clinical or diagnostic data on individual patients, but only the respective *Plasmodium* species and the outcome [59–64]. They are only considered in the following if they are explicitly mentioned. One of these cases has recently been published in detail and was included in our analysis [65, 66].

Table 1 gives an overview of the most important data from 57 patients (the 5 patients treated in our departments and 52 additional case reports from the literature). The complete dataset including the 23 cases from overview articles is available as Supplementary material.

**Table 1:** Overview of selected clinical and diagnostic criteria of the cases from the literature [1–51] and the cases treated in our departments [52–56]

No	Year	Author	Age	Sex	Country of treatment	Place/mode of infection	PF	PV	PO	HLH-2004 criteria (≥ 5!)	HScore	HScore likelihood (%)	Treatment HLH	Complications	Outcome
1	1995	Anwar	"Young"	M	Pakistan	Pakistan	x			3	130–274	5–> 99	None		Recovered
2	1996	Ohno	24	M	Japan	"Tropical travel"	x			5	130–319	5–> 99	None		Recovered
3	2000	Aoubah	41	F	France	Costa Rica		x		5	178	54–70	None		Recovered
4	2000	Retornaz	73	M	France	Madagascar	x			3	190–239	70–99	IVIG		Recovered
5	2000	Sermet-Gaudelus	2	M	France	Gabon	x			4	180–299	54–> 99	PL		Recovered
6	2002	Al-Kilani	25	M	Saudi Arabia	Saudi Arabia	x			3	134–244	9–> 99	IVIG		Recovered
7	2002	Zvulunov	11	F	Israel	Cameroon	x	x		4	130–309	5–> 99	None		Recovered
8	2003	Park	23	M	South Korea	South Korea		x		4	150–294	16–99	None		Recovered
9	2004	Abdelkefi	25	M	Tunisia	Blood transfusion	x			4	158–321	25–> 99	None		Recovered
10	2004	Saribeyoglu	3	M	Turkey	Turkey				5	228–277	96–> 99	None		Recovered
11	2007	Ohnishi	30	F	Japan	Papua New Guinea	x			3	78–270	1–> 99	MPL, PL, plasma exchange	AKI	Recovered
12	2008	Niang	37	F	Senegal	Senegal	x			4	109–230	1–98	MPL	AKI	Recovered
13	2009	Albaker	37	F	Saudi Arabia	Nepal		x		5	155–190	25–80	None		Recovered
14	2010	Dass	16	M	India	India	x			6	288	> 99	MPL, PL	Shock, ARDS, Coma	Recovered
15	2010	Ram Kumar	9	F	India	India		x		6	223–253	96–> 99	None		Recovered
16	2011	Bae	22	M	South Korea	South Korea		x		4	149–243	16–> 99	None		Recovered
17	2011	Vinoth	11 Mon	M	India	India	x			5	235	98–99	None		Recovered
18	2011	Sung	64	F	South Korea	South Korea		x		5	160	25–40	None		Recovered
19	2012	Klein	47	M	France	Cameroon	x			4	150–237	16–99	None		Recovered
20	2012	Rehman	22	M	Saudi Arabia	.	x			5	251–319	> 99	None		Recovered
21	2012	Sanklecha	12	F	India	India	x			5	165–278	40–> 99	None		Recovered
22	2013	Trapani	6	M	Italy	Togo	x			5	261–312	> 99	MPL		Recovered
23	2013	Tanwar	"Child"	F	India	India		x		6	249	> 99	None		Recovered
24	2013	Mukharjee	21	F	India	India		x		5	224–269	96–99	MPL		Recovered
25	2013	Padhi	1	M	India	India		x		4	124–214	5–96	None		Recovered
26	2014	Pothapregada	8	F	India	India		x		5	215	93–96	None	Shock, Coma	Recovered
27	2014	Trifi	16	F	Tunisia	Ivory coast	x			6	239–284	98–> 99	IVIG	ALI, AKI	Recovered
28	2015	Bhagat	5	F	India	India	x	x		5	239	98–99	PL		Recovered
29	2015	Santos	5	M	Portugal	Mozambique	x			6	249–299	> 99	None		Recovered
30	2015	Khurram	19	M	Pakistan	Pakistan		x		5	250	> 99	DX, MPL	ARDS, DIC, Shock	**†**
31	2016	Ullah	20	M	Pakistan	Pakistan		x		5	107	1–3	None		Recovered
32	2016	Weeratunga	55	M	Sri Lanka	South Africa	x			6	274–303	> 99	None	Coma	Recovered
33	2016	Bhatia	4	M	Nepal	Nepal		x		6	230	96–98	None	Coma	Recovered
34	2017	Jaiswal	8	M	India	India	x			6	228–258	96–> 99	None		Recovered
35	2017	Ladeb	27	M	Tunisia	Blood transfusion	x			4	161–312	40–> 99	MPL, PL		Recovered
36	2017	Muthu	43	M	India	India	x	x		6	243	> 99	IVIG	ARDS	Recovered
37	2017	Muthu	34	F	India	India	x			5	261	> 99	IVIG	ARDS	Recovered
38	2017	Selvarajan	24	M	India	India	x			6	219–283	93–> 99	DX		Recovered
39	2017	Kumar	29	F	India	India		x		5	164–218	40–96	None		Recovered
40	2017	Harioly Nirina	32	F	Madagascar	Madagascar	x			6	174–238	54–99	None		Recovered
41	2018	Amireh	23	M	USA	Nigeria		x		6	193–228	80–98	None	AKI, Coma	Recovered
42	2019	Cheikhrouhou	62	M	Tunisia	Airport	x			6	214–273	93–> 99	None	MOF	**†**
43	2020	Crestia	13	M	France	Airport	x			4–5	206–311	86–> 99	None		Recovered
44	2020	Sharma	7	F	India	India		x		6	249	> 99	None		Recovered
45	2020	Srivatsav	3	M	India	India	x			5	> 250	> 99	DX		Recovered
46	2021	Chaudhry	6	F	India	India	x			6	233–283	98–> 99	None		Recovered
47	2021	Chaudhry	11	M	India	India	x			4	160–283	25–> 99	None		Recovered
48	2021	Zhou	29	F	China	Nigeria	x			7	249	> 99	MPL, IVIG	ALI, AKI, ARDS, DIC	Recovered
49	2022	Almajed	32	F	US	Djibouti	x			5	150–204	16–93	None		Recovered
50	2022	Sadek	5	M	GB	Nigeria	x			5	202–289	88–> 99	DX	ALI, AKI, Shock, DIC	Recovered
51	2022	Havvat	37	M	Turkey	Sudan		x		6	166–239	40–99	IVIG	DIC	Recovered
52	2018/2023	Rojo-Marcos	58	F	Spain	Angola			x	6	253	> 99	MPL, IVIG		Recovered
53^a^	2017, unpublished		35	M	Germany	Togo	x			4	229–279	96–> 99	None	AKI	Recovered
54^a^	2019, unpublished		42	F	Germany	Cameroon	x			4	244	> 99	DX, IVIG		Recovered
55^a^	2019, unpublished		48	M	Germany	Uganda	x			5	268	> 99	DX, plasmapheresis	MOF	**†**
56^a^	2020	Fuchs	33	M	Germany	Nigeria	x			7	217	93–96	DX, etoposide, ruxolitinib	DIC, AKI, ALI	Recovered
57^a^	2022, unpublished		31	F	Germany	Sierra Leone	x			7	279	> 99	DX	ARDS	Recovered

*Mon.* months, *PF* *Plasmodium falciparum*, *PV* *Plasmodium vivax*,..: no data, *PL* prednisolone, *MPL* methylprednisolone, *DX* dexamethasone, *IVIG* intravenous immunoglobulin, *AKI* acute kidney injury, *ARDS* acute respiratory distress syndrome, *ALI* acute liver injury, *DIC* disseminated intravascular coagulation, *MOF* multiple organ failure

^a^Patient treated in our departments

In 55 of the 57 included cases, the patients’ age was recorded, ranging from 11 months to 73 years (median 23 years, IQR 8.5–34.5). Thirty-four patients were male and twenty-three were female. In 47 patients for whom data were available, the median delay between the onset of malaria symptoms and hospital admission (patient delay) was 7 days (IQR 5.5–12.5). Ethnicity of patients was available in only ten cases: five were Caucasian, four African, and one mixed African/Caucasian. Most patients have been treated in malaria-endemic countries in Asia (*n* = 48; 60%; including the overview articles), mostly India (26) and Thailand (6). Twenty-one malaria infections had been acquired in Sub-Saharan Africa, nineteen of the patients were travelers or migrants who were later diagnosed in their home countries.

Including our own cases and the cases from overview articles, most case reports describe infections with *Plasmodium falciparum* (55/80; 69%), followed by *Plasmodium vivax* (26/80; 33%) including co-infections with both *Plasmodium* species (4/80; 5%) and *Plasmodium ovale wallikeri* (1/80; 1%). In two cases, the *Plasmodium* species was unspecified. No HLH cases were attributed to *Plasmodium malariae* or *Plasmodium knowlesi*. Data on parasitaemia are available for 24 of the 38 cases with *P. falciparum*, with 11 (46%) exceeding a parasite load of 5%. In five cases, malaria was not the only infection diagnosed: co-infection with Dengue virus is mentioned in two cases [35, 48], and *Mycobacterium tuberculosis*, *Mycoplasma pneumoniae* or HIV plus HCV in one case each [26, 38, 40]. Two cases were diagnosed as airport malaria, and two cases were transfusion associated after bone marrow stem cell transplant [17, 44–46]. These four cases occurred outside endemic countries.

Overall, HLH-2004 criteria were met in 40 out of the collected 57 cases (70%), as far as documentation is available. Some of the cases have been published before the most recent revision of diagnostic criteria in 2007. In some other cases, current criteria were reportedly met, but not all relevant values were accessible.

Some cases were published before the introduction of HScore in 2014. HScore was mentioned in only one publication [48]. However, we retrospectively calculated HScore for all published cases. Unfortunately, only 18 of the 57 case reports (32%) included the complete data sets required for the calculation. In all other cases, we calculated the minimum and maximum possible value for the HScore, assuming that either no additional criteria or all criteria were met. Table 2 shows details on HLH-2004 criteria and additional HScore criteria. The available data show that 37 cases (65%) have an HScore of more than 169 points. Only two patients (4%) have a lower score, but interestingly, both met five HLH-2004 criteria. For the remaining 18 patients, values above or below 169 are possible depending on the missing data. The median HScore of cases with complete data sets is 244. In patients who received specific HLH therapy, the median HScore was 250, while patients without HLH therapy had a median HScore of 223. According to the Mann–Whitney *U* test, the difference was significant with a *p* value < 0.05. However, the individual diagnostic criteria (temperature, platelet count, hemoglobin, neutrophil count, triglycerides, fibrinogen, ferritin, sCD25, AST, spleen length) did not show any significant difference between the treated and the untreated group. Two of the patients treated in our departments fulfill only four HLH-2004 criteria but have an HLH probability of over 90% according to HScore. One of these patients did not have a bone marrow biopsy, while the other patient's bone marrow biopsy showed hemophagocytosis.

**Table 2:** Frequency of positive HLH-2004 criteria and additional HScore criteria in patients treated or untreated for sHLH

HLH criteria	Frequency (*n* positive/*n* examined; %)	Median (IQR)	Median treated (IQR)	Median untreated (IQR)	*p* value	Remarks
Fever	55/55; 100%	39.4 °C (39.0–40.0)	39.7 (39.0–40.0)	39.2 (39.0–39.5)	0.18	Exact temperature available in 34 cases
Splenomegaly	46/47; 98%	Spleen length: 138 mm (120–161)	141 (137–171)	120 (120–138)	0.71	Spleen length available in only 11 cases, rest diagnosed clinically or no data
Platelets < 100/nl	53/56; 95%	40/nl (28–59)	34 (22–51)	51 (31–63)	0.3	Exact numbers available in 53 cases
Hemoglobin < 90 g/l	44/56; 79%	74 g/l (60–89)	70 (60–86)	76 (56–93)	0.57	Exact numbers available in 52 cases
Neutrophils < 1/nl	15/29; 52%	1.035/nl (0.808–1.381)	1.276 (0.957–1.730)	0.9 (0.8–1.2)	0.10	In another 17 cases, total WBC was documented (3/nl; 2.325–3.8)
Triglycerides (≥ 265 mg/dl)	34/44; 77%	303 mg/dl (268–428)	314 (272–428)	306 (260–405)	0.55	
Fibrinogen < 1.5 g/l	17/31, 55%	1.29 g/l (0.9–2.16)	1.51 (1.10–2.48)	1.06 (0.8–1.47)	0.97	3 values excluded due to lack of plausibility
Ferritin > 500 µg/l	51/52; 98%	2165 µg/l (942–5919)	4,446 (2164–8465)	1,000 (817–3094)	0.35	Exact number available in 41 cases; in 7 more cases, ULD was exceeded. Since ULD varied between 500 and 100,000, these figures were not used for median calculation
CD25 > 2.400 U/ml	6/7; 86%	4352 U/ml (3489–13,217)	13,217 (5183–20,803)	3677 (3489–4014)	0.21	Available in six cases only
Impaired NK cell activity	0/1; 0%					Available in one case only
Typical histology	42/44; 95%					In one case, hemophagocytosis detected in peripheral blood
Immunosuppression	3/57; 5%					No immunosuppression was assumed if not explicitly mentioned
Hepatomegaly	29/38; 76%					Exact values from imaging available in five cases only
Elevated AST	34/37; 92%	147 U/l (95––315)	208 (103–446)	130 (85–192)	0.23	Exact values available for 28 patients

Severe complications were described in 20 out of 57 cases (35%), including acute kidney injury (AKI, *n* = 9), acute respiratory distress syndrome (ARDS, *n* = 7), somnolence/coma (*n* = 6), acute liver injury (ALI, *n* = 5), shock (*n* = 5), disseminated intravascular coagulation (DIC, *n* = 5), and unspecified multiple organ failure (MOF, *n* = 1). In three cases, fatal outcome was reported following multiple organ failure. One of these patients had suffered from co-infection with Dengue virus and *Plasmodium vivax* malaria [35], a second patient was diagnosed with airport malaria caused by *P. falciparum* late in the course of the disease [45]. The third patient suffered a fulminant course only 2 days after the onset of symptoms. The other 54 detailed reports relate full recovery of the patients, mostly without specific treatment for HLH. Only 24/57 (42%) patients received treatment for HLH, mostly corticosteroids (*n* = 18; 75%), intravenous immunoglobulins (IVIG) (*n* = 9; 38%), including both (*n* = 3; 13%). One of the patients received a salvage therapy comprising dexamethasone, etoposide, and ruxolitinib [8], another one was treated with dexamethasone and plasmapheresis.

Case series mentioning HLH because of malaria report 2 more patients successfully treated with IVIG and another 15 positive outcomes without treatment details. One patient with cerebral malaria died, although it is not clear whether sHLH was decisive in the fatal outcome. The other five cases contained no data on treatment or outcome either.

The Destatis query yielded three cases with ICD-10 codes corresponding to a combination of malaria and HLH. These cases match three of our patients with HLH diagnosed during hospital stay within the years 2016–2020. The fourth patient was diagnosed only retrospectively, and the corresponding ICD-10 code was not documented during his hospital stay. The fifth patient was diagnosed outside the period of available Destatis data. The register data make the existence of additional cases unlikely.

## Discussion

We present the largest cases series (*n* = 5 cases) and the most comprehensive literature research (*n* = 52 cases) of sHLH triggered by malaria. In most cases, further diagnostics were performed due to persistent fever despite proven absence of plasmodia, which eventually led to the diagnosis of sHLH. The most frequently met criteria for HLH diagnosis were fever (100%), elevated ferritin (98%), splenomegaly (98%), hemophagocytosis (95%), thrombocytopenia (95%), and elevated AST (92%). Malaria and sHLH, however, share diagnostic and clinical features which include fever, anemia, low platelets, elevated AST, as well as an increase of biomarkers like ferritin and sCD25, which makes even uncomplicated malaria meet several diagnostic criteria of HLH. Even hemophagocytosis is not mandatory for diagnosing sHLH, and it may not be specific either since it has also been observed in cases of uncomplicated malaria [67]. Thus, applying the HLH-2004 criteria in patients with malaria may result in a high number of rather improper sHLH diagnoses, which emphasizes the need for more specific diagnostic criteria for HLH secondary to malaria. Furthermore, in many of the case reports analyzed, the HLH-2004 criteria were not assessed and cannot be assessed retrospectively because the required data are incomplete. The validity of the HScore has been proven in different constellations, with an overall sensitivity and specificity comparable to HLH-2004 when adjusting the cutoff [68, 69]. To test the validity for malaria patients, we calculated the HScore for our patients and for cases from the literature. As complete data were only available for 18/57 patients, a reliable evaluation is not possible. Interestingly, however, HScore results in patients requiring HLH therapy (median 250) differed significantly from those with spontaneous resolution of symptoms (median 223). It is, therefore, at least conceivable that a higher cutoff value for malaria patients could enable better prediction of the clinical course.

Of the five sHLH patients treated in our departments, four had a serious life-threatening course, requiring specific HLH therapy including one case with a ruxolitinib-containing salvage therapy. One patient was treated with plasmapheresis, while another one was diagnosed with sHLH more by chance and did not require specific therapy. This is consistent with the data from the other 52 cases in the literature, of whom 32 (62%) received no additional treatment for sHLH. Even of the 20 patients with documented severe complications, 5 recovered without specific HLH therapy and only 3 died, of whom 1 was co-infected with Dengue virus. The second patient had suffered from a febrile illness for a month until malaria was diagnosed, as malaria was not endemic in his country and had not been considered as a possible cause of his symptoms for a long time. Whether the Dengue co-infection or the delayed antimalarial treatment was decisive for development of sHLH remains speculative, however. The third patient, who had been treated in one of our departments, had allegedly noticed symptoms only 2 days before admission and deceased from multiple organ failure only 2 days after hospitalization. In this case, additional underlying conditions could not be ruled out, as a post-mortem was not conducted. A further statistical delimitation of risk factors for a fatal course was not feasible. The case fatality rate (CFR) is calculated as 5% (4/75) if all cases published so far with available outcome data (i.e., including the overview articles) are considered. An older study reported a CFR for infection-associated HLH of up to 52%, with the subset of HLH due to EBV infection having the poorest prognosis. Although the overall prognosis has improved with newer treatment strategies, recent studies have described mortality in sHLH due to EBV infection as high as 25%, even with optimal treatment [6]. In contrary, sHLH due to Parvovirus B19 has been reported to have a favorable outcome in eight out of eleven reportedly healthy individuals (73%) treated with corticosteroids only or no treatment at all [70]. A review of sHLH cases caused by *Mycobacterium tuberculosis* from 1975 to 2014 reports a CFR of 49% (31/63) with increased mortality due to lack or delay of antimycobacterial therapy [71]. A recent review of sHLH triggered by visceral leishmaniasis, the most frequently reported protozoal disease to cause sHLH, reports favorable outcome in 45 out of 54 cases (83%), of whom only 29 (54%) had received specific treatment for HLH. In many of the included cases, a delayed diagnosis of visceral leishmaniasis was noted [72]. In comparison, our data suggest a fairly good prognosis or probably an over-diagnosis of malaria-associated sHLH.

In contrary to the risk of over-diagnosing, huge numbers of malaria-associated sHLH may remain undiagnosed, when considering that many diagnostic criteria for HLH require advanced laboratory capacities and that the clinical syndromes overlap significantly. Since the majority of patients recovered from sHLH following antimalarial therapy only, it may be assumed, that sHLH as defined by available diagnostic tools has a high chance to remain undetected. In vivax malaria, thrombocytopenia has been recognized a rather uncommon feature [73] and has been suggested as an indicator for complicated courses by Antinori et al. [74]. While some of the cases mentioned in their review have been diagnosed with sHLH, thrombocytopenia may just have been one feature of an eventually missed diagnosis of sHLH in other cases.

Some authors hypothesize, that sHLH usually subsides when its trigger is eliminated by effective antimalarial therapy but may continue as self-sustaining mechanism once a tipping point is exceeded, like in sepsis or other hyperinflammatory disorders. In this context, it is interesting that microscopic clearance of *Plasmodium* spp*.* does not necessarily result in immediate clearance of their antigens: HRP-2, for example, a protein often targeted in rapid tests for *P. falciparum*, can be detected for several weeks after successful therapy [75]. Although HRP-2 may not necessarily be the trigger for HLH, other antigens may also remain in the bloodstream for a longer period, and thus maintain the immune response.

The median delay in seeking healthcare after beginning of malaria symptoms (patient delay) of 7 days (IQR 5.5–12.5) for patients with malaria-associated sHLH is more than twice as high as the 3 days (IQR 2–6) reported in a recent meta-analysis of malaria patients overall in non-endemic countries [76]. In another study with 1181 cases of falciparum malaria, the median time from symptom onset to malaria treatment was 4 days (IQR 2–7) [77]. For lack of data, the diagnostic delay, defined as duration between symptom onset and diagnosis, cannot be extracted from most case reports. Interestingly, the published case reports contain a high number of untypical routes of infection (two transfusion associated cases, two cases of airport malaria), which often lead to delayed diagnosis of malaria. Thus, the available data suggest that prolonged parasitemia may increase the risk of sHLH, which goes in line with an increased risk of sHLH due to delayed treatment reported for other infectious diseases such as tuberculosis and visceral leishmaniasis [71, 72]. While no reports are yet available for malaria, other infectious agents such as *Parvovirus B19* and *Leishmania infantum* have been shown to cause recurrent sHLH in case of relapsing or persistent infection [78, 79], supporting the tipping point hypothesis after prolonged or repeated exposure.

In the defined study period of 8 years, we were able to document 5 cases of sHLH in a total of 1461 malaria cases (0.34%) treated in the participating centers. We assume, that the motivation to participate in the study was lower in the centers that had not made any sHLH diagnosis, and that thus, the frequency among the rest of the 6,682 reported malaria cases in Germany during the study period may be lower. The Destatis data with only three documented cases of HLH and malaria within the same hospital stay confirm this assumption. However, even if no additional cases occurred during the 8 years, the percentage of patients developing HLH following plasmodia infection in Germany would be as high as 0.07%. In 2021, the WHO estimated a total of 247 million cases and 619 thousand fatal cases of malaria worldwide. Sub-Saharan Africa accounts for 95% of all malaria cases worldwide with *P. falciparum* being the most prevalent *Plasmodium* species [80], while only 2 of the 52 previously published sHLH cases (4%) were reported from malaria-endemic countries in Africa. It is difficult to transfer our figures to endemic areas, since partial immunity of the continuously exposed population may have influence on the development of hyperinflammatory conditions such as HLH. This may also explain the high number of reports from Asia, where the predominantly seasonal and less-frequent transmission may cause stronger inflammatory responses. On the other hand, insufficient diagnostic resources may at least partly account for the comparatively few reports from Sub-Saharan Africa. Although it remains highly speculative, transferring the frequency of 0.07% to the situation in Africa would result in about 175 thousand cases of sHLH per year following malaria infections.

## Conclusion

Most published cases of HLH triggered by malaria resulted in favorable outcome despite eschewal of specific HLH therapy; overall consequences may, therefore, seem less daunting. Nevertheless, some of the reported complicated cases would probably have been fatal if left untreated. It is, therefore, reasonable to assume that a proportion of fatalities in malaria patients is due to undiagnosed sHLH.

According to the reviewed literature and the treatment data of our patients, a long course of *Plasmodium* infection preceding antimalarial treatment appears to increase the risk of HLH. Persistent or relapsed fever despite *Plasmodium* clearance—as observed in four out of the five patients treated in our departments—should give reason to rule out sHLH.

Interestingly, some patients who meet the HLH-2004 criteria do not meet the HScore criteria and vice versa. This, and the frequent spontaneous resolution of HLH symptoms following antimalarial treatment, suggest that ultimately both scores with currently applied cutoff values are not suitable for diagnosing malaria-induced sHLH with adequate certainty.

Thus, in a low resource setting, empirical corticosteroid therapy may remain a pragmatic approach for prolonged febrile disease despite parasite clearance, when individually balanced against risk of side effects and possible underlying co-infections.

## Limitations

The included case reports differed considerably in terms of scope, as well as diagnostic and therapeutic approach. Due to the heterogeneity, a combined analysis is generally problematic, a multivariate analysis of risk factors was not possible at all. Nevertheless, we decided to include all available data to increase the validity. Some cited sources contain incomplete data sets; therefore, calculation of the HScore or verification of HLH-2004 criteria is limited. For the HScore, this is indicated by a range of numbers. Especially in cases with large ranges, validity cannot be assessed.

The risk assessment is based on a very low number of cases in a non-endemic setting and can, therefore, only serve as an orientation.

### Supplementary Information

Below is the link to the electronic supplementary material.

Supplementary file1 (XLSX 33 kb)

## Data Availability

All relevant data have been made available within the manuscript or as supplementary material.
